# An in vitro model to assess the immunosuppressive effect of tick saliva on the mobilization of inflammatory monocyte-derived cells

**DOI:** 10.1186/s13567-015-0229-5

**Published:** 2015-09-28

**Authors:** Nathalie Vachiery, Carinne Puech, Patricia Cavelier, Valérie Rodrigues, Rosalie Aprelon, Thierry Lefrançois, Dominique Martinez, Mathieu Epardaud

**Affiliations:** INRA-CIRAD, UMR 1309 Contrôle des maladies animales, exotiques et émergentes, F-34398 Montpellier, France; INRA-CIRAD, UMR 1309 Contrôle des maladies animales, exotiques et émergentes, F-97170 Petit-Bourg, Guadeloupe France; UMR C5535 Institut de Génétique Moléculaire de Montpellier, Montpellier, France; INRA, UMR 1282 Infectiologie et Santé Publique, 37380 Nouzilly, France

## Abstract

Tick-borne pathogens cause potent infections. These pathogens benefit from molecules contained in tick saliva that have evolved to modulate host innate and adaptive immune responses. This is called “saliva-activated transmission” and enables tick-borne pathogens to evade host immune responses. Ticks feed on their host for relatively long periods; thus, mechanisms counteracting the inflammation-driven recruitment and activation of innate effector cells at the bite site, are an effective strategy to escape the immune response. Here, we developed an original in vitro model to evaluate and to characterize the immunomodulatory effects of tick saliva that prevent the establishment of a local inflammatory immune response. This model mimics the tick bite and enables the assessment of the effect of saliva on the inflammatory-associated dynamic recruitment of cells from the mononuclear phagocyte system. Using this model, we were able to recapitulate the dual effect of tick saliva on the mobilization of inflammatory monocyte-derived cells, i.e. (i) impaired recruitment of monocytes from the blood to the bite wound; and (ii) poor mobilization of monocyte-derived cells from the skin to the draining lymph node. This simple tool reconstitutes the effect of tick saliva in vivo, which we characterized in the mouse, and should enable the identification of important factors facilitating pathogen infection. Furthermore, this model may be applied to the characterization of any pathogen-derived immunosuppressive molecule affecting the establishment of the inflammatory immune response.

**Electronic supplementary material:**

The online version of this article (doi:10.1186/s13567-015-0229-5) contains supplementary material, which is available to authorized users.

## Introduction

Ticks transmit a variety of disease-causing infectious agents of medical and veterinary importance. Ticks are second only to mosquitoes as vectors of agents causing diseases in humans, and they are the most important arthropod that transmits pathogens to other animal species. Tick saliva contains numerous pharmacologically-active molecules that modulate host hemostasis, wound healing, pain and itch responses and inflammation. These tick-induced changes in host defenses enable extended blood feeding and hence facilitate pathogen transmission. In arthropod-borne diseases, saliva is critical for potent infection because it contains molecules with potent immunomodulatory properties that favor the evasion of host immune responses. This is called “saliva-activated transmission” (SAT) and restricts protective immune responses against vector-borne pathogens [1]. The saliva and/or salivary gland extract (SGE) of ixodid (hard) adult tick species can suppress or redirect host innate and specific acquired immune responses. Some of the immunomodulatory strategies used include impairment of T-lymphocyte function, suppression and deviation of the production and action of cytokines and alteration of the function of antigen presenting cells (APCs). Indeed, some molecules contained in saliva bind to numerous cytokines and hence suppress the activity of various immune cells (reviewed in [2]).

Local inflammation and the inflammation-driven recruitment of innate cells must be contained for the pathogen to escape the immune response during extended feeding. Blockade of inflammatory cells affects local immunity but also limits the potential establishment of the adaptive response.

Dendritic cells (DC) are widely accepted to be the most potent APCs in the immune system because of their strong capacity for acquiring and processing antigens for presentation to T-cells and their potential for driving T-cell activation [3,4]. DCs and other APCs from the *mononuclear phagocyte system* play a pivotal role in the activation of the adaptive immune response [5]; therefore, these cells are frequently targeted by pathogens suppressing immune responses.

Hard ticks have thus evolved strategies to overcome this arm of the host immune defense. Some molecules, such as prostagalandin E2 (PGE2), adenosine and Sialostatin L, have broad-spectrum immunosuppressive activity and affect a variety of host immune cells [6,7]. Moreover, two modulators, Salp15 and Japanin specifically suppress the functions of DCs. The Salp15 protein modulates CD4^+^ T-cell function but also inhibits cytokine secretion by DCs [8,9]. The Japanin protein, which was recently isolated by chromatograpy from *Rhipicephalus appendiculatus*, exerts a potent immunosuppressive effect on DCs by altering their maturation and therefore their activity [10].

Finally, the Evasin proteins, which have been characterized in the saliva of several tick species, are potent anti-inflammatory agents that efficiently block various chemokines [11,12]. In particular, Evasin-1 blocks the CCL3-mediated adhesion and migration of leukocytes and thus affects the mobilization of various leukocytes, including APCs, during the immune response [11,12].

Here, we examined how tick saliva affects important steps of the development of an effective immune response. In particular, we focused on the recruitment and activation of monocyte-derived macrophages and inflammatory DCs in “damaged-skin”. We used a modified form of the reverse-transmigration model to reconstitute the principal preliminary steps of infection in vitro. This model is a useful in vitro technique originally designed to evaluate the in vivo differentiation of macrophages and DCs from monocytes in blood. This method mimics the recruitment of monocytes from peripheral blood to the infected tissue where they can either stay and give rise to macrophages, or enter into lymphatic vessels, a process inducing their maturation into DC (Mo-DC), en route to the draining lymph node, which is the site of the initiation of the adaptive immune response, while monocyte are commonly considered to access to the draining lymph by the high endothelial venules after blood transport [13,14]. Thus, to understand better the mechanisms by which SAT alters the recruitment of macrophages, Mo-DCs and other APCs, we established an in vitro strategy based on the dynamic reverse-transmigration model. With this model, we were able to reproduce accurately the dual immunosuppressive effect of tick saliva that we first observed in vivo in the mouse. We found that tick saliva impaired both the recruitment of Mo-DC and macrophage precursors from the blood to the tick bite and the migration of potential Mo-DCs from this site to the draining lymph nodes. This is a useful method to screen in vitro for molecules with immunosuppressive or immunostimulatory effects on macrophages and Mo-DCs mobilization under inflammatory condition.

The identification and characterization of the factor(s) present in tick saliva that may interfere with inflammatory-derived MPS cells and innate and adaptive immune responses is important for the development of strategies to prevent pathogen transmission.

## Materials and methods

### Ethics statement

All animal experiments were conducted according to internationally recognized OIE standards and approved by the director of the veterinary services of Guadeloupe on behalf of the prefecture of Guadeloupe in August 2006 (authorization number: A−971−18−01). Certificates of authorization are available upon request. The owners of the Creole goats gave permission for their animals to be used in this study, which was carried out in strict accordance with the recommendations of the ethics committee for animal experimentation of Languedoc Roussillon, France. The study was registered at the “Comité National de Réflexion éthique sur l’Experimentation Animale” (CNREEA) under the registration code: C2EA-36.

### Tick saliva preparation

To collect saliva, adult female *Amblyomma variegatum* ticks were allowed to feed for 10 to 15 days on the backs of naïve Creole goats originating from Les Saintes islands, according to instructions from the Guide to the Care and Use of Experimental Animals provided by the French Ministry of Agriculture. Partially engorged female ticks were removed and fixed by their dorsum on sticky paper. To stimulate salivation, engorged ticks were injected to the dorsa with 5 μL of pilocarpine solution 5% (w/v) in PBS, pH 7.4, using a 0.3 mL 30 gauge 13 mm insulin syringe (Becton Dickinson, Franklin Lakes, NJ, USA). Saliva was harvested using a microcapillary, kept on ice, pooled, centrifuged through a 0.22 mm pore filter (Costar-Corning Inc., Cambridge, MA, USA) and stored at −80 °C until further use. Three pools of saliva were used for the experiments and each saliva pool consisted of material harvested from 30 to 50 semi-engorged female ticks. The saliva protein concentration was determined by the BCA assay (Procedure TPRO-562; Sigma Chemical Co., St. Louis, MO, USA).

### Suturing of mice and fluorescein isothiocyanate (FITC) painting protocol

C57BL/6 mice (6–8 weeks of age) were purchased from Charles River and then housed at the Institut de Génétique Moléculaire de Montpellier (IGMM). A combination of two approaches was used to mimic the tick bite; (1) a nonspecific inflammatory stimulus caused by a silk monofilament surgical suture [15,16] positioned on the back of the mouse, to reproduce the physical damage of the rostrum; and (2) an intradermal injection within the suture area of either a control solution (5% pilocarpine in PBS) or tick saliva, once a day for 3 days. The concentration of pilocarpine used (5%) is an estimation of the maximal final concentration of pilocarpine in saliva, considering that Ribeiro et al. estimate that its concentration is potentially ≤1% in other ixodes species [17]. The same aliquot of PBS-pilocarpine was used for mock intradermal injection in mice to control for any potential contamination of tick saliva during the collection process. After the final injection, the sutured area was painted with a mix of FITC in acetone:dibutylphtalate (1:1). This solution stains all cells in the suture area, which enabled cells migrating from this area to the draining lymph nodes (i.e. axillary and brachial) to be tracked. Draining lymph nodes were excised 24 h later to evaluate the effect of saliva on the mobilization of DCs and other APCs. Distal lymph nodes (inguinal) were collected as a non-draining control. Cells were then isolated and processed for flow cytometry as described below. Skin tissues from control and sutured areas were cut into sections in a cryotome, and the sections were counterstained with hematoxylin to visualize leukocyte infiltrates. The institutional review board of the local animal facility approved all protocols in accordance with national guidelines.

### Isolation and culture of bovine aortic endothelial cells (BAECs)

Primary isolation: BAECs were isolated from segments of descending thoracic aorta obtained from a local slaughterhouse. The aortas were rinsed briefly in sterile PBS to remove free blood and transported to the laboratory in ice-cold PBS supplemented with antibiotics (100 μg/mL penicillin, 100 U/mL streptomycin, and 50 μg/mL Fungizone). To release endothelial cells, the lumen of the aorta was incubated with collagenase (1 mg/mL, Eurobio) in PBS for 15 min at 37 °C and was washed with BHK21 complete media (Eurobio). After centrifugation, the cell pellet was resuspended in BHK21 complete media with antibiotics and plated at a concentration of approximately 1 × 10^4^ cells/cm^2^. Subcultures were prepared using trypsin-EDTA (0.01% trypsin, 0.02% EDTA) and potential BAEC “islets” were selected to discard contaminating fibroblasts. BAEC were characterized by their morphology and by positive immunofluorescent staining with an antibody against human factor VIII (von Willebrand factor) (DAKO), detected with an anti rabbit-FITC-conjugated secondary antibody. Cells were used between passage 3 and 6.

### In vitro reverse-transmigration protocol

BAECs, 1 × 10^6^ cells per well, are cultured in BHK21 complete media, on top type 1 collagen-coated Biocoat 24 well plates with a 3 μm transwell (Becton Dickinson, Franklin Lakes, NJ, USA). For experiments implicating zymosan, zymosan A from saccharomyces cerevisiae (InVivoGen) was added at 0.005% w/v to the bottom of the transwell prior to BAEC addition. After 2 to 3 days, BAEC reach confluency and total bovine peripheral blood mononuclear cells (PBMCs), 2 × 10^6^ cells per well, are added on top of the BAEC monolayer. After 1 hour, most of the monocyte from the PBMC have already migrated threw the BAEC and the top of the transwell is washed 2 time with HBSS containing 2 mM EDT to remove left over cells that have not transmigrated. After HBSS/EDTA was of the top of the transwell, classical microscopy observation shows no trace of left over PBMC. 48 h later, the top and bottom fraction of the transwell are collected in HBSS/EDTA to characterize the transmigrated cells.

### Flow cytometry

Mice lymph nodes were excised, sliced and gently digested at 37 °C for 1 h using collagenase D, 1 mg/mL (Roche, Basel, Switzerland) and passed on Nitex filters in PBS containing 5% FCS to obtain single cell suspensions. After counting, lymph node cells were incubated with R-phycoerythrin (PE)-coupled CD11b (M1/70 clone) mAb, PerCP-coupled CD8a (53–6.7 clone) mAb, PE-Cy7-coupled F4/80 (6 F12 clone) mAb, Allophycocyanine (APC)-coupled Gr1 (RB6-8C5) mAb, Horizon V450-coupled CD11c (HL3 clone) mAb and APC-eFluor 780-coupled B220 (CD45R, RA3-6B2 clone) mAb (BD PharMingen, San Diego, CA, USA) in PBS containing 2% FCS and 0.02% sodium azide for 30 min at 4 °C. After washing, cells were analyzed on a FACSCanto II cell analyzer using Diva software (BD, Mountain View, CA, USA). Bovine cells collected from reverse-transmigration protocols were stained with the indicated primary mouse anti-bovine mAbs. MHC class II (HLA-DR, TH14B clone) and CD86 (B7-2, ILA190A clone) mAbs were obtained from VMRD, Pullman, Washington. The SIRPα (CD172a, CC149 clone) mAb was obtained from Serotec, Kidlington, UK. Primary mAbs were detected by secondary staining with FITC-, PE- or Tricolor-conjugated anti-mouse isotype-specific Abs (Serotec, Kidlington, UK).

### Statistical analysis

**Figure 1:**
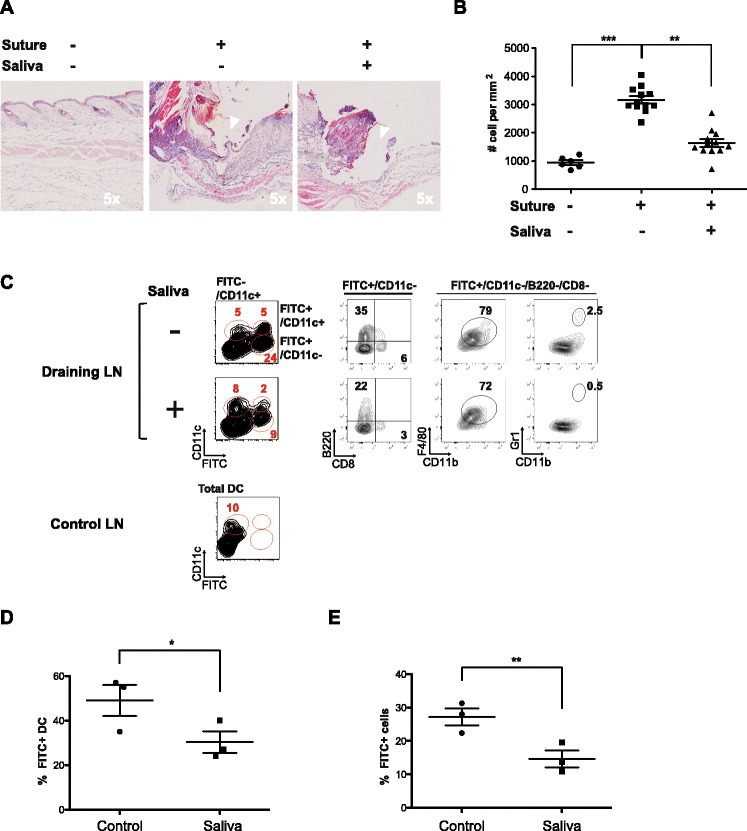
**Dual effect of tick saliva in vivo on the recruitment of leukocyte infiltrates to skin and dendritic cells to draining lymph nodes.** A surgical monofilament suture was positioned on the back of a C57BL/6 mouse and tick saliva or a control solution was intradermally injected once a day for 3 days into the suture area. The skin at the sutured area was then stained with FITC solution and mice were killed 24 h later to analyze the skin and lymph nodes. Data were obtained from two independent experiments including three mice per condition. (**A**) Representative hematoxylin and eosin staining (H&E) of histological sections of skin biopsies. **B** Density of infiltrated leukocytes in skin biopsies was estimated by counting the total number of nuclei within histological samples of skin sections, of similar size and including the sutured-damaged area, using Image J software (two mice per condition, 10 sections per mouse). Statistically significant differences between groups were assessed with the ANOVA (Kruskal-Wallis) test and Dunn’s multiple comparison post test, * P < 0.05 and ** P < 0.005. **C** Contour plots of resident DCs (FITC^−^CD11c^+^), migrating DCs (FITC^+^ CD11C^+^) and other migrating cells (FITC^+^ CD11C^−^) in the draining lymph nodes from a control skin area (no FITC, Control LN) and from a sutured areas injected with saliva or control solution (Draining LN). FITC^+^ CD11C^−^ cells include migrating B cells (B220^+^), macrophages (CD11b^+^F4/80^+^) and granulocytes (CD11b^+^Gr1^+^). **D**-**E** The relative proportion of total migratory cells (FITC^+^) and migratory DCs (FITC^+^ CD11C^+^) in lymph nodes draining the suture area was determined in the absence or presence of saliva. The mean percentage of migratory cells in each condition is indicated with a line and each point represents one mouse. Statistically significant differences between the two groups were determined by the Student’s t test, *P < 0.05 and **P < 0.005.

The statistical significance of the results shown in Figure 1B was determined using the Kruskal-Wallis test and the Dunn’s multiple comparisons post-test. The statistical significance of the results shown in all other figures was determined using the Student’s *t* test with a one-tailed distribution and a two-sample test assuming equal variance. In all statistical analyses, data were considered to be statistically different (*) at *P* < 0.05.

## Results

### Tick saliva impairs leukocyte infiltration in skin lesions

The establishment and maintenance of effective immune surveillance in skin relies on (1) the recruitment of immune cells to the site of the skin lesion driven by inflammatory stimuli, and (2) the subsequent migration of APCs to the skin draining lymph node. We used a C57BL/6 mouse model to evaluate in vivo the effect of the tick bite on these two critical events, in particular focusing on the mobilization of mononuclear phagocytes and polymorphonuclear cells and the migration of DCs and other APCs.

In this in vivo model, we reproduced the two main characteristics of a tick bite: physical damage caused by the rostrum was mimicked by a monofilament surgical suture positioned on the back of the mouse, and the suture area was injected intradermally once a day for 3 days with either saliva extracted from *Amblyomma variegatum* or a control solution to model the biochemical effects of saliva. The skin tissue was then processed 24 h after the final injection to quantify the leukocyte infiltrates within the suture area (white arrows in Figure 1A-5x indicate area damaged by the suture).

Counts of local leukocytes were significantly higher in the sutured area than in non-damaged skin (*P* < 0.0001) as assessed by a *Kruskal-Wallis test and a Dunn’s multiple comparison post test* (Figures 1A and B). On the other hand, in the suture area injected with tick saliva, counts of leukocytes dropped down significantly (*P* < 0.001) being 50% lower than in the area injected with the control solution (Figure 1B). A closer look at the infiltrate surrounding the suture area, in the absence of saliva, shows cells displaying a typical morphology with large round mononuclear cells characteristic of monocytes or macrophages and large round cells with multilobed nuclei characteristic of neutrophils (Additional file 1-Zoom 40x, white arrow).

Although we can not exclude that saliva might affect the rate of cell death or proliferation, the absence of cell death induction in vitro (Additional file 2) strongly suggest that the major effect of saliva is the impairment of the recruitment of inflammatory immune cells to damaged skin, which may affect the initiation of the immune response.

### Tick saliva impairs the migration of APCs to the skin draining lymph node

Next, we investigated the effect of tick saliva on the migration of sentinel cells to the skin draining lymph node. After the final saliva (or mock) injection on day 3, the sutured area was painted with a FITC staining solution (acetone:dibutylphtalate [1:1]) to label cells confined to the biting area and the draining lymph node was excised 24 h later. This is a common method to follow cell migration through lymph from the skin to the draining lymph node, although we cannot exclude minimal interference from cell-free FITC taken up by lymph node resident cells [18,19]. Nevertheless, this protocol enables the evaluation, within draining lymph nodes (brachial and axillary), of the migration of DCs and other APCs from damaged skin following inflammation caused by the suture. We used dibutylphtalate, which sensitizes the skin and significantly promotes the migration and accumulation of cells within the lymph node but without affecting the relative proportion of the principal APCs subtypes, as shown in Additional file 3.

Flow cytometry analysis revealed that the percentage of DCs (CD11c^+^FITC^+^, Mig DC) migrating to the skin draining lymph nodes was 50% lower in mice injected with saliva than in those injected with the control solution, (23.4 ± 7% vs. 45 ± 11%, respectively) (Figures 1C and D). In terms of absolute cell number, 19.2 × 10^4^ ± 2 × 10^4^ DCs were present in draining lymph nodes in the absence of saliva, whereas only 6.3 × 10^4^ ± 2.5 × 10^4^ were present following injection with saliva. This observation is consistent with previous studies showing that *Ixodes Ricinus* tick saliva impairs the early migration of DCs to draining lymph nodes [20]. If DCs are considered essential for the induction of immune response we must highlight that we observed a notable impairment of percentage of CD11c^−^FITC^+^ cells, which outnumbers the CD11c^+^FITC^+^ cells, to draining lymph nodes in the presence of saliva. These CD11c^−^FITC^+^ cells correspond in majority to other non-DC potential antigen carrying and presenting cells, the most part represented by macrophages and activated monocytes (CD11b^+^F4/80^+^) and B cells (B220^+^), with the addition of few granulocytes (CD11b^+^Gr1^+^) (Figure 1C). Thus, the percentage of total migratory cells was almost 50% lower in mice injected with tick saliva than in those injected with control solution (27.6% +/− 4.1 to 14.1% +/− 4.4) (Figures 1D and E). However we cannot evaluate the level of cell free FITC migration, which might be minimal considering the use of soluble and not dextran-associated FITC, could affect this result. Moreover, besides the possibility of resident APCs to pick-up free migrating FITC we should also consider the possibility that some resident APCs might acquire the FITC after phagocytosis of other migrating APCs.

Thus, our in vivo results show that tick saliva has a dual immunosuppressive effect on inflammatory responses in skin. First, tick saliva impairs the infiltration of leukocytes including mononuclear cells and neutrophils from the blood. This effect probably involves the recently described anti-chemokine proteins (Evasins) identified in the extracts of tick salivary glands [12]. Second, tick saliva strongly inhibits the mobilization of APCs from the bite site to the draining lymph nodes.

### An adapted version of the reverse-transmigration model recapitulates the recruitment of bovine blood monocytes and the subsequent mobilization of monocyte-derived mononuclear phagocytes upon tissue inflammation

The reverse-transmigration protocol, previously described by Randoph et al. [21], is based on the in vivo biology of inflammatory monocyte-derived DCs, in particular their mobilization and maturation from blood precursors (i.e. monocytes). This model takes advantage of the maturation that occurs when monocytes are recruited from the blood to an inflamed peripheral tissue, where they counteract infection by giving rise to macrophages but also to DC precursors that subsequently pass through lymphatic vessels, maturing en route to the draining lymph nodes. In the original culture system, blood mononuclear cells (PBMCs) are deposited on a monolayer of human umbilical vein endothelial cells (HUVECs) grown on collagen [21]. After only one hour of co-culture, monocytes reach the collagen by traversing the endothelial monolayer in a luminal-to-abluminal direction. This transmigration process reflects the extravasation of monocytes from the blood to an infected skin area. Within 48 hours of culture, approximately 50% of the transmigrated monocytes migrate back through the endothelium in an abluminal-to-luminal direction. This second reverse-transmigration step mimics the migration of DC precursors from the peripheral tissue to the lymphatic vessels connecting to the draining lymph node. This second, “backward”, migration event, despite the use of a blood instead of a lymphatic endothelium layer, is sufficient to trigger the maturation of the reverse-transmigrated cells from monocytes to DCs [21-23].

**Figure 2:**
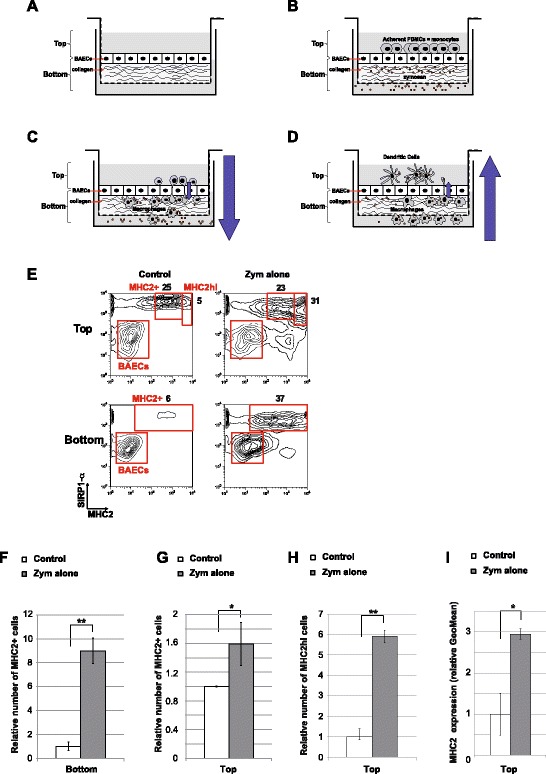
**Adaptation of the reverse-transmigration in vitro model with bovine cells to recapitulate monocyte-derived cells mobilization from blood to the draining lymph node under inflammatory condition.** **A**-**D** Schematic representation of the in vitro differentiation of blood monocytes into macrophages and Mo-DCs using the reverse-transmigration model. **E**-**I** Two-color flow cytometry assay of monocyte-derived cells collected from monocyte/BAEC cultures grown on collagen-coated transwells according to the reverse-transmigration protocol. Non-adherent and low-adherent cells within the top and bottom sections of the transwell were processed for cytometry analysis. Left contour plots show the conditions in the absence of any exogenous stimulus whereas right contour plots show conditions with zymosan. Cells were labeled with anti-SIRP1-α to discriminate monocyte-derived cells from endothelial cells. The expression of MHCII within the SIRP1-α^+^ population was also determined to discriminate within monocyte-derived cells potential macrophages (MHCII^+^) and potential Mo-DC (MHCII^hi^) (**E**). The percentages of SIRP1-α^+^MHCII^+^, SIRP1-α^+^MHCII^hi^ and BAECs in each representative dot plot are indicated (**E**). **F** Comparison showing the relative number of SIRP1-α^+^MHCII^+^ cells in the bottom of the transwell in conditions with or without zymosan stimulation. **G** Comparison showing the relative number of SIRP1-α^+^MHCII^+^ cells in the top section of the transwell in conditions with or without zymosan stimulation. **H** Comparison showing the relative number of SIRP1-α^+^MHCII^hi^ cells in the top section of the transwell in conditions with or without zymosan stimulation. **i** Comparison showing the relative expression level of MHCII in SIRP1-α^+^MHCII^+^ cells in the top section of the transwell in conditions with or without zymosan stimulation. **F**-**I** The value of the control condition was arbitrarily set to 1. Data are presented as the mean ± SD based on four independent experiments each including at least three transwells per condition. Statistically significant differences between the two groups were determined by the Student’s t test, *P < 0.05 and **P < 0.005.

In addition to the in vitro production of monocyte-derived DCs (Mo-DC), we hypothesized that this model is also useful to investigate the dynamic of local inflammatory responses, in particular those involving monocyte-derived mononuclear cells. Therefore, we adapted the reverse-transmigration protocol to evaluate monocyte recruitment and the mobilization of monocyte-derived mononuclear phagocytes under inflammatory conditions. We used bovine cells because *Amblyomma variegatum* also targets cattle. Primary bovine aorta endothelial cells (BAECs) were grown to confluence in a monolayer on top of a collagen-coated transwell (Figure 2A). Zymosan was added as an immunostimulus to the bottom of the transwell and bovine PBMCs, which are the main source of blood monocytes, were deposited on top of the BAEC monolayer 24 h later (Figure 2B). Non-adherents PBMCs were removed by several washing after 1 to 2 h, to remove any microscopically visible left over PBMC on top of the BAEC, but most had already migrated from the topical to the basal side of the BAEC monolayer (i.e. luminal-to-abluminal transmigration, Figure 2C). These migrating cells mimic the extravasion of monocytes from blood to the inflamed tissue (Figure 2C). When they reached the collagen layer, most transmigrating monocytes acquired a more mature phenotype reminding of macrophages. However, within the next 48 hours, some monocytes, which were in close contact with the basal pole of BAECs, were able to re-transverse the endothelial barrier and differentiate into more mature cells with a phenotype resembling monocyte-derived DCs upon the uptake of zymosan (i.e. abluminal-to-luminal reverse-transmigration, Figure 2D). This latter step recapitulates the migration of Mo-DC precursors from the inflamed tissue to the draining lymphatic vessels. Thus, the collection of cells from the top or bottom of the transwell enables the quantitative and qualitative assessment of two critical steps of monocyte-derived mononuclear cells mobilization.

We analyzed the expression of SIRP1-α, which is specific of myeloid lineage cells and also expressed by some lymphatic migrating DC subset [24-26], to discriminate mononuclear cells from residual BAECs. As previously described for human cells [21], we confirmed that transmigration and reverse-transmigration without stimuli naturally occur at low levels but are strongly stimulated by the addition of zymosan to the abluminal side (Figures 2E, F, G and H).

Regarding the first step, i.e. transmigration (Figure 2C), the number of transmigrating monocytes was almost ten-fold higher in the presence than in the absence of zymosan (Figures 2E (bottom) and F). Furthermore, flow cytometry revealed 40 ± 4.7% of SIRPα^+^MHCII^+^ transmigrated cells (Figure 2F (bottom), Zym alone) in the presence of zymosan whereas this proportion was only 4.5 ± 1.6% in the absence of zymosan (Figure 2E (bottom), Control).

Regarding the second step, i.e. reverse-transmigration (Figure 2D), we detected two distinct subsets of reverse-transmigrated cells, including one subset exhibiting a potential phenotype of maturing Mo-DCs with a stronger expression of MHCII (Figure 2E (top), MHCII^hi^) [27]. The proportion of total SIRP1-α^+^MHCII^+^ cells accessing the top fraction was slightly higher in the presence than in the absence of zymosan (Figures 2E (top) and F) (48 ± 9% vs. 30 ± 0.2%, respectively) (Figure 2E (top)); however, the addition of zymosan resulted in the substantial expansion of the MHCII^hi^ subset of reverse-transmigrated cells, with almost six times more SIRP1-α^+^MHCII^hi^ in the presence of zymosan than in control conditions (Figures 2E (top) and H) with a global increase of the relative level of MHCII expression (Figure 2I). Given previous findings of the reverse transmigration model in human cells [21-23], and the high expression level of MHCII, it is possible that the SIRP1-α^+^MHCII^hi^ cell subset correspond to monocytes maturing into closed to Mo-DCs. Furthermore, the SIRP1-α negative cell subset may also contain some cells corresponding to maturing DCs because SIRP1-α negative DCs have been identified in pseudo-afferent lymph collected from sheep and cattle [24,25,28].

Nevertheless, our results are consistent with observations made on human blood-derived monocytes transmigrated on HUVECs [21] and thus validate the potential of this system to recapitulate, under inflammatory conditions, the mobilization and differentiation of bovine blood monocytes into APCs sharing characteristics of macrophages and potential Mo-DCs. Thus, this is a useful model to assess in vitro the dynamic effect of saliva, or any immunomodulatory compound, on the maturation and migration of monocyte-derived cells under inflammatory condition.

### The reverse-transmigration in vitro model recapitulates the dual effect of tick saliva on the mobilization of monocyte-derived mononuclear phagocytes

We next assessed whether the in vitro reverse-transmigration model could be used to evaluate the effect of tick saliva on the recruitment of monocyte-derived phagocytes. We thus performed reverse-transmigration experiments with zymosan, in the absence or presence of tick saliva and compared these findings with those obtained in vivo in mice (Figure 1).

**Figure 3:**
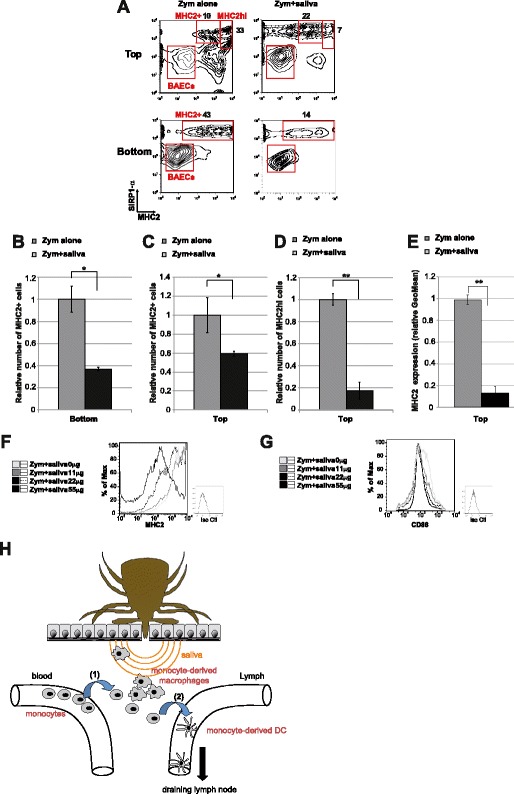
**In vitro characterization of the dual immunosuppressive effect of tick saliva on inflammatory-induced mobilization of monocyte-derived mononuclear phagocytes.** **A**-**E** Two-color flow cytometry assay of monocyte-derived cells collected from monocyte/BAEC cultures grown on collagen-coated transwells for 48 h according to the reverse-transmigration protocol. Left contour plots show conditions with zymosan alone whereas right contour plots show conditions with zymosan and tick saliva. **B** Comparison showing the relative number of SIRP1-α^+^MHCII^+^ cells in the bottom of the transwell in conditions with zymosan alone or with zymosan and saliva. **C** Comparison showing the relative number of SIRP1-α^+^MHCII^+^ cells in the top section of the transwell in conditions with zymosan alone or with zymosan and saliva. **D** Comparison showing the relative number of SIRP1-α^+^MHCII^hi^ cells in the top section of the transwell in conditions with zymosan alone or with zymosan and saliva. **E** Comparison showing the relative expression level of MHCII in SIRP1-α^+^MHCII^+^ cells in the top section of the transwell in conditions with zymosan alone or with zymosan and saliva. **B**-**E** The value for the control condition (zymosan alone) was arbitrarily set to 1. Data are presented as the mean ± SD based on four independent experiments each including at least three transwells per condition. Statistically significant differences between the two groups were determined by the Student’s t test, *P < 0.05 and **P < 0.005. (**F**&**G**) Representative histograms of MHCII and CD86 staining on SIRPα^+^ monocyte-derived cells incubated for 48 h with zymosan and saliva at amounts varying from 0 to 55 μg/mL. One representative experiment out of two independent experiments is shown. **G** Schematic representation of the effect of tick saliva on the mobilization of monocyte-derived APCs in vivo. (1) Monocytes from the blood are recruited to the area of the tick bite where a proportion of the cells differentiate into potential macrophages. (2) Potential Mo-DC precursors then migrate from this area into the draining lymphatic vessels.

The number of transmigrated cells was 50% lower in the presence of saliva and zymosan than in control conditions of zymosan alone, with only 14.8 ± 0.6% of cells migrating to the bottom well following the addition of saliva (Figures 3A (bottom) and B). Observation at an early time point, i.e. 4 h, reveal an impairment of the initial migration when saliva is added to zymosan, suggesting that the 50% reduction of transmigration, observed at 48 h, is mainly the consequence of the reduction of the first transmigration by saliva (Additional file 4, zymosan vs zymosan + saliva).

Furthermore, the total number of MHCII^+^ cells that reverse-transmigrated to the top of the BAEC monolayer was about 40% lower in the presence of saliva and zymosan than in conditions of zymosan alone, with only 28.6 ± 1.3% of total SIRP1-α^+^MHCII^+^ cells migrating back to the top in the presence of saliva (Figures 3A (top) and C). Moreover, saliva almost completely abrogated the mobilization of SIRP1-α^+^MHCII^hi^ cells by reverse-transmigration, with only 5.4 ± 2.5% of these cells migrating back through the endothelial layer in the presence of saliva and zymosan compared with 32 ± 1.7% in the presence of zymosan alone (Figure 3A (top)). This striking difference of more than 80% in the number of MHCII^hi^ reverse-transmigrating cells indicates that saliva strongly impairs the zymosan-induced recruitment of potential Mo-DCs (Figures 3A (top) and D). These in vitro findings are consistent with our in vivo experiments showing that tick saliva inhibits the mobilization of MPS cells from the bite wound to the draining lymph node in mice (Figures 1C and D). Moreover, the addition of different concentrations of saliva to zymosan influenced both the recruitment of monocyte-derived cells (data not shown) and their maturation, as shown by the negative correlation between saliva concentration and the expression of MHCII and the costimulatory molecule CD86 on the cell surface (Figures 3E, F and G). Notably, the addition of tick saliva, even at high doses, did not significantly affect the viability of the cells collected in the bottom or top of the transwell (as shown respectively in Additional files 2A and B).

Thus, this in vitro method is able to recapitulate some aspects of the effects observed in vivo (Figure 1). Overall, these findings show that tick saliva counteracts the inflammatory-associated immune response by affecting both the recruitment of blood mononuclear cells to the bite wound and the subsequent mobilization of monocyte-derived phagocytes from this site to the draining lymph node.

## Discussion

Here, we used an in vivo mouse model and an in vitro migration assay to examine the effect of tick saliva on inflammatory cells. Our in vivo experiments reveal that tick saliva substantially impairs the mobilization of mononuclear cells to the bite wound and limits the subsequent migration of APCs to the draining lymph node, the site of the initiation of the adaptive immune response. Similarly, our in vitro model also revealed the dual effect of tick saliva on the local inflammatory related immune response. Consistent with our in vivo findings, tick saliva impaired the recruitment of blood monocytes in vitro but also prevented their maturation in situ into monocyte-derived macrophages and potential Mo-DCs during abluminal-luminal migration (Figure 3H). Thus, this model clearly demonstrates that saliva strongly influences the tramsmigration of monocytes (Figure 2C and Figure 3H (1)); however, it is possible that inhibition of reverse-transmigration (Figure 2D and Figure 3H (2)) is a consequence of the low numbers of potential Mo-DC precursors, which in turn results from their impaired recruitment from blood.

These findings suggest that molecules secreted in saliva affect both the recruitment of monocytes from blood to the bite site and their subsequent migration from the bite site to the draining lymph node. The chemokine-blocking Evasin proteins, affecting notably CCL3, are probably involved in the inhibition of the recruitment of monocytes from the blood [12] (Figure 3H (1)). In addition, the Japanin protein inhibits the differentiation of monocytes into Mo-DCs and may also inhibit the maturation Mo-DCs and the mobilization of these cells from the inflamed area to the lymphatic system [10] (Figure 3H (2)). Our in vitro model is a useful approach for the isolation and analysis of immunomodulatory compounds affecting local inflammation-induced recruitment of macrophages and the maturation and migration of Mo-DCs. Indeed, classical in vitro protocols based on bone marrow or monocyte-derived macrophages or DCs [29], requiring 7 days of cytokine-supplemented culture and are not suitable for analyzing the dynamic aspects of these APCs. Nonetheless, the migration of macrophages and the mobilization and maturation of Mo-DCs are key features in the establishment of an appropriate immune response.

The in vitro reverse-transmigration model is a valuable method to analyze the effect of an immunosupressive agent, such as tick saliva, on the recruitment, differentiation and activation of macrophages and Mo-DCs. One limitation of this in vitro model is that it focuses only on inflammatory recruited blood-derived macrophages and Mo-DCs and not on tissue-resident DCs. Despite this limitation, the long feeding period of ixodid ticks (i.e. several days) requires the neutralization of inflammatory precursors from the blood [30], which justifies the rationale of evaluating the effect of saliva on blood-derived APCs. This technique will lead to the isolation and identification of new immunomodulatory agents from complex mixes of proteins, and hence the discovery of new agents.against tick-borne pathogens. Furthermore, classical (static) or simple transmigration (one way) in vitro protocols cannot evaluate the dynamics of macrophages and DC precursors and their mobilization to the lymphatic system. Thus, the reverse-transmigration protocol is a valuable tool for the identification and characterization of potential immunosuppressors or adjuvants capable of affecting the biology of monocytes, macrophages and DCs. Thus, this model is a suitable alternative to the use of an in vivo model and can be used for high-throughput screening to identify potentially immunomodulatory molecules.

## Additional files

Additional file 1:
**Morphology of infiltrated leukocytes next to the suture position within skin biopsies.** Representative hematoxylin and eosin staining (H&E) (10X & 40X) of histological sections of skin biopsies close to the localization of the surgical monofilament coinjected with control solution (t = 24 h). Some infiltrated cells displayed a typical morphology with large round mononuclear cells characteristic of monocytes or macrophages and some display a multilobed nuclei characteristic of neutrophils (Zoom 40x, white arrow). Additional file 2:
**Effect of increasing saliva concentrations on total cell viability in the reverse transmigration transwell protocol.** The viability of SIRP1-α^+^ monocyte-derived cells collected from monocyte/BAEC cultures grown on collagen-coated transwells according to the reverse-transmigration protocol was assessed following 48 h of incubation with zymosan and saliva at amounts varying from 0 to 55 μg/mL. (A) Relative number of dead SIRP1-α^+^ cells in the bottom of the transwell. (B) Relative number of dead SIRP1-α^+^ cells in the top of the transwell. Additional file 3:
**Effect of dibutylphtalate on the recovery of FITC** ^**+**^ **cells in draining lymph nodes.** Comparison showing the relative proportion of total migratory cells, migratory DCs (CD11c+) and migratory B cells (B220^+^) in lymph nodes draining the suture area in the absence or presence of dibutylphtalate in the FITC painting solution. Additional file 4:
**Early kinetic of bovine monocyte transmigration threw BAECs.** Two-color flow cytometry assay of monocyte-derived cells collected from the bottom of monocyte/BAEC collagen-coated transwells 2 h post-PBMC deposition on the BAEC monolayer. Left contour plots show conditions without zymosan or saliva (Control) middle contour plots show condition with zymosan alone whereas right contour plots show conditions with zymosan and tick saliva.

## Abbreviations

APC: antigen presenting cell
BAECs: bovine aortic epithelial cells
Mo-DC: monocyte-derived dendritic cell
Mφ: macrophage
MPS: mononuclear phagocyte system
HUVECs: human umbilical vein endothelial cells
