# Supplementary figures and images for: An in vitro model to assess the immunosuppressive effect of tick saliva on the mobilization of inflammatory monocyte-derived cells
Source: Vet Res. 2015 Sep 28;46:117. doi: 10.1186/s13567-015-0229-5 (PMC4586012; doi:10.1186/s13567-015-0229-5)

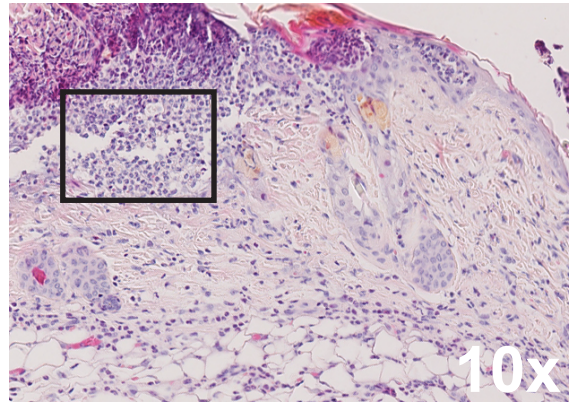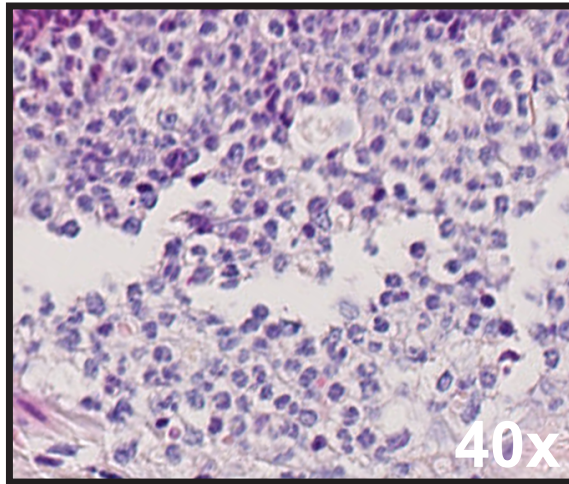

Supplement: Additional file 1: — Morphology of infiltrated leukocytes next to the suture position within skin biopsies. Representative hematoxylin and eosin staining (H&E) (10X & 40X) of histological sections of skin biopsies close to the localization of the surgical monofilament coinjected with control solution (t = 24 h). Some infiltrated cells displayed a typical morphology with large round mononuclear cells characteristic of monocytes or macrophages and some display a multilobed nuclei characteristic of neutrophils (Zoom 40x, white arrow). [file 13567_2015_229_MOESM1_ESM.pdf]

**A**

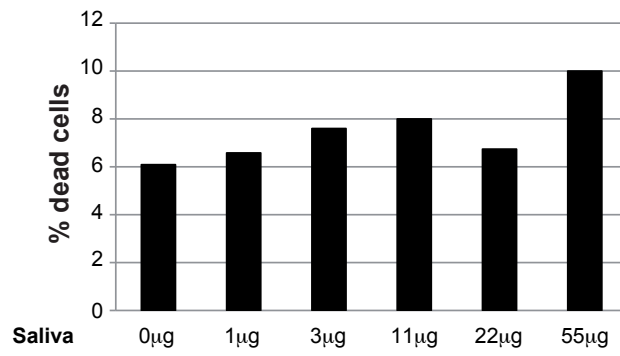

**B**

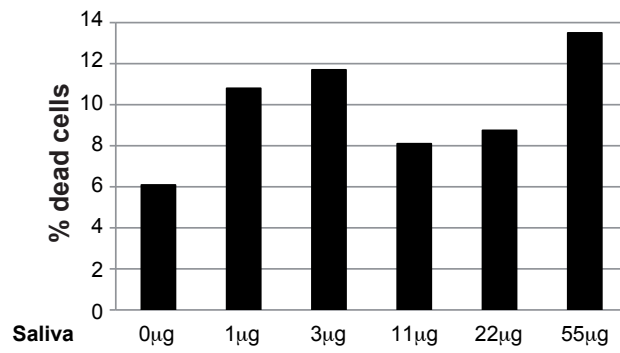

Supplement: Additional file 2: — Effect of increasing saliva concentrations on total cell viability in the reverse transmigration transwell protocol. The viability of SIRP1-α+ monocyte-derived cells collected from monocyte/BAEC cultures grown on collagen-coated transwells according to the reverse-transmigration protocol was assessed following 48 h of incubation with zymosan and saliva at amounts varying from 0 to 55 μg/mL. (A) Relative number of dead SIRP1-α+ cells in the bottom of the transwell. (B) Relative number of dead SIRP1-α+ cells in the top of the transwell. [file 13567_2015_229_MOESM2_ESM.pdf]

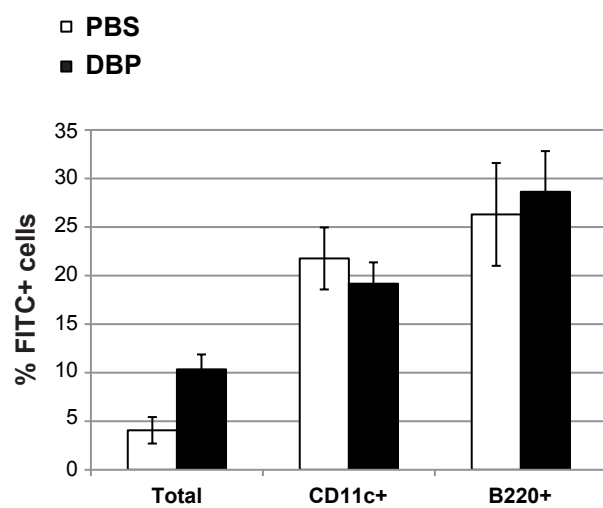

Supplement: Additional file 3: — Effect of dibutylphtalate on the recovery of FITC + cells in draining lymph nodes. Comparison showing the relative proportion of total migratory cells, migratory DCs (CD11c+) and migratory B cells (B220+) in lymph nodes draining the suture area in the absence or presence of dibutylphtalate in the FITC painting solution. [file 13567_2015_229_MOESM3_ESM.pdf]

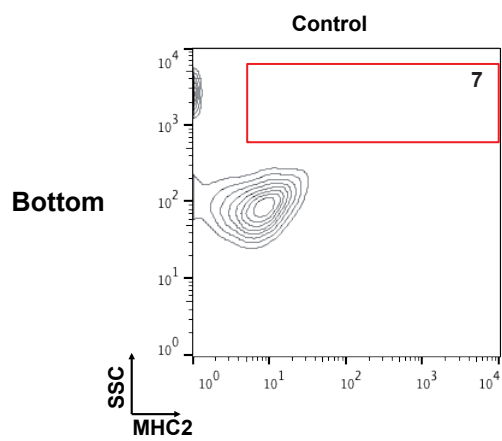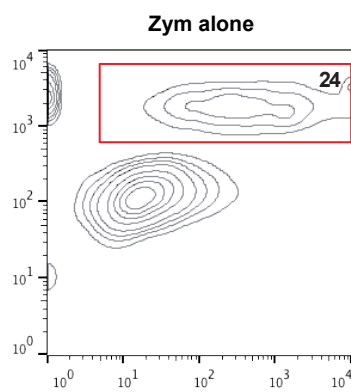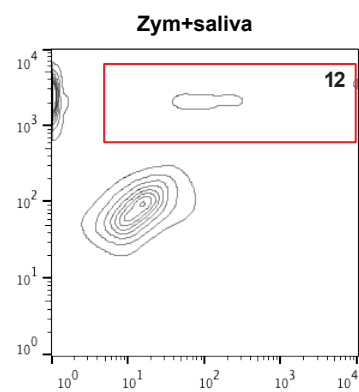

Supplement: Additional file 4: — Early kinetic of bovine monocyte transmigration threw BAECs. Two-color flow cytometry assay of monocyte-derived cells collected from the bottom of monocyte/BAEC collagen-coated transwells 2 h post-PBMC deposition on the BAEC monolayer. Left contour plots show conditions without zymosan or saliva (Control) middle contour plots show condition with zymosan alone whereas right contour plots show conditions with zymosan and tick saliva. [file 13567_2015_229_MOESM4_ESM.pdf]
